# The mitochondrial genome of *Apis mellifera siciliana*

**DOI:** 10.1080/23802359.2022.2073844

**Published:** 2022-05-10

**Authors:** Dora Henriques, Cecília Costa, José Rufino, Maria Alice Pinto

**Affiliations:** a Centro de Investigação de Montanha (CIMO), Instituto Politécnico de Bragança, Bragança, Portugal; b Centro di Ricerca Agricoltura e Ambiente (CREA-AA), Consiglio per la Ricerca in Agricoltura e l’Analisi dell’Economia Agraria, Bologna, Italy; c Research Centre in Digitalization and Intelligent Robotics, Instituto Politécnico de Bragança, Bragança, Portugal

**Keywords:** Mitogenome, *Apis mellifera siciliana*, African subspecies, phylogenetic relationship

## Abstract

We assembled the mitogenome of *Apis mellifera siciliana,* which was previously identified as African by the tRNA-leu-cox2 intergenic region. The mitogenome is 16,590 bp long. The gene content and organization are identical to other *A. mellifera* mitogenomes, containing 13 protein-coding genes, 22 transfer RNA genes, and 2 ribosomal RNA genes. Phylogenetic analysis showed a close mitochondrial relationship between *A. m. siciliana* and other African subspecies such as *Apis mellifera sahariensis*, *Apis mellifera intermissa*, and *Apis mellifera ruttneri.*

*Apis mellifera siciliana* Dalla Torre 1896 is a honey bee subspecies autochthonous to Sicily (Italy; Ruttner 1988). While morphological and microsatellite markers place *A. m. siciliana* in the East European evolutionary lineage, the intergenic tRNA-leu-cox2 mitochondrial DNA region assigns it to the African lineage (Ruttner 1988; Garnery et al. 1993; Franck et al. 2000; Muñoz et al. 2014). Recently, the native genetic background of this subspecies has been changed by human-mediated gene flow, when the Sicilian beekeepers started to import *Apis mellifera ligustica* (Longo 1984). In an attempt to protect the genetic integrity of the native gene pool, which has been threatened by introgressive hybridization with *A. m. ligustica*, conservation programs were implemented in small islands of the *A. m. siciliana* native range, like Filicudi and Vulcano (Muñoz et al. 2014). The aim of this work is to describe the mitogenome of a Sicilian specimen previously identified as A2 by Henriques et al. (2019) from sequencing data of the tRNA-leu-cox2 intergenic region.

The specimen was deposited at Centro de Investigação de Montanha, Instituto Politécnico de Bragança (http://cimo.ipb.pt/cimo/web/index.php?r=site/about, M. Alice Pinto, apinto@ipb.pt) under the voucher number 3512. The sample is from a haploid male collected in 2013 in the municipality of Termini, province of Palermo (Latitude: 37.9601 N, Longitude 13.7229 E) by the beekeeper Carlo Amodeo. The DNA was extracted from the thorax using a phenol/chloroform isoamyl alcohol (25:24:1) protocol (Sambrook et al. 1989). Whole-genome sequencing was accomplished using the Illumina HiSeq 2500 platform. Prior to sequencing, libraries were generated using the Illumina TruSeqTM Sample Preparation kit. The 2 × 150 paired-end sequence reads were assembled and annotated by MitoZ v2.3 (Meng et al. 2019). The obtained mitogenome was reordered considering the reference genome NC_001566 and the annotation was manually adjusted in MEGA X (Kumar et al. 2018). The sequences of the 13 protein-coding genes were concatenated from 26 mitogenomes (the new one from *A. m. siciliana* and 25 available on GenBank) and used for the phylogenetic analysis (Figure 1). The tree was inferred using the maximum likelihood method implemented in MEGA X using 1000 replicates (Kumar et al. 2018).

**Figure 1.**
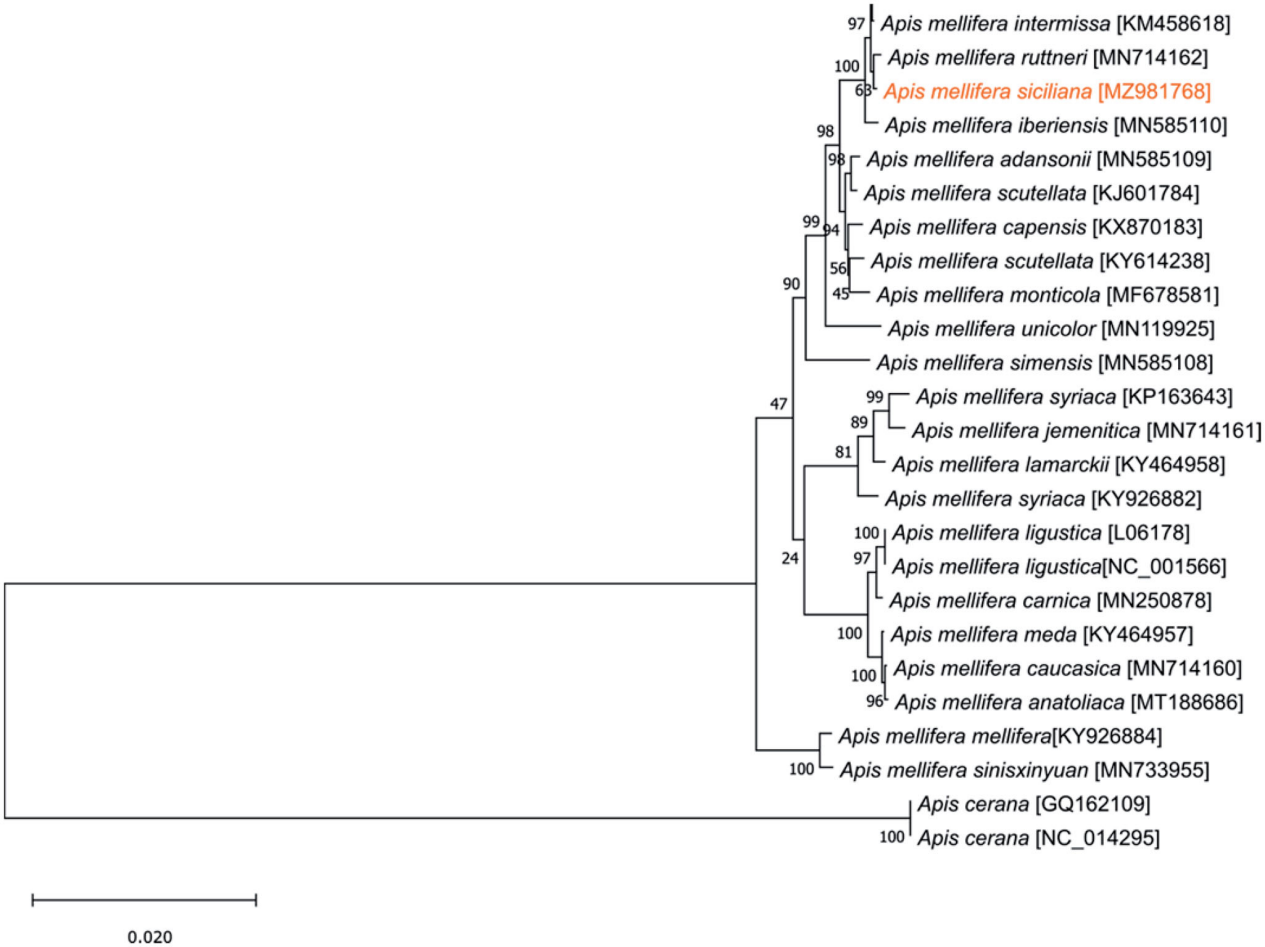
Phylogenetic tree showing the relationship between *Apis mellifera siciliana* (marked in orange) and other 23 *Apis mellifera* subspecies. *Apis cerana* was used as an outgroup. Numbers at the nodes indicate the percentage of trees in which the associated taxa clustered together. GenBank accession number is listed under brackets after the species and subspecies names.

The obtained mitogenome is 16,590 bp long (Accession Number MZ981768), with an overall base composition of 45.9% T, 37.1% A, 8.7% C, and 8.3% G. Similar to other *A. mellifera* mitogenomes, the *A. m. siciliana* mitogenome has 13 protein-coding genes, 2 RNA genes, and 22 tRNA genes. The size of the 13 protein-coding genes ranged from 159 bp (*ATP8*) to 1665 bp (*ND5*). Most of the protein-coding genes (*ND2*, *COX1*, *COX2*, *ATP8*, *ATP6*, *COX3*, *ND3*, *ND6*, and *CYTB*) and 14 tRNAs (*tRNA-Glu*, *tRNA-Ser*, *tRNA-Met*, *tRNA-Gln*, *tRNA-Ala*, *tRNA-Ile*, *tRNA-Trp*, *tRNA-Leu*, *tRNA-Asp*, *tRNA-Lys*, *tRNA-Gly*, *tRNA-Asn*, *tRNA-Thr*, and *tRNA-Ser*) are encoded in the light strand. In contrast, the other four protein-coding genes (*ND1*, *ND4*, *ND4L*, and *ND5*), eight tRNAs (*tRNA-Cys*, *tRNA-Tyr*, *tRNA-Arg*, *tRNA-Phe*, *tRNA-His*, *tRNA-Pro*, *tRNA-Leu*, and *tRNA-Val*), 16S rRNA (1321 bp; 83.6% AT) and 12S rRNA (827 bp; 82.0% AT) are encoded in the heavy strand. The size of the 22 tRNAs ranged from 63 bp (*tRNA-Ser* and *tRNA-Gln*) to 78 bp (*tRNA-Thr*). ATP6 and ATP8 share a common 19-bp long fragment (from the position 4663 bp to 4681 bp), specifically the 3′ end of ATP8 coincides with the 5′ end of ATP6. Concerning the start codons, six genes started with ATT (*COX2*, *ND5*, *ND4L*, *ND6*, *ND1*, and *ATP8*), four with ATG (*ATP6*, *COX3*, *CYTB*, and *ND4*), two with ATA (*COX1* and *ND3*) and one (*ND2*) with ATC. All protein-coding genes share the stop codon TAA.

Notably, *A. m. siciliana* forms a tight cluster in the phylogenetic tree with the North African subspecies *Apis mellifera sahariensis* and *Apis mellifera intermissa*, with the *Apis mellifera ruttneri* from Malta and with *Apis mellifera iberiensis* from the Iberian Peninsula, suggesting a recent shared ancestry among these subspecies. Placement of *A. m. siciliana* in the African lineage result is congruent with the maternal structure already known for this subspecies and with the information provided with tRNA-leu-cox2 intergenic region lineage (Ruttner 1988; Garnery et al. 1993; Franck et al. 2000; Muñoz et al. 2014; Henriques et al. 2019).

## Data Availability

The genome sequence data that support the findings of this study are openly available in GenBank of NCBI at https://www.ncbi.nlm.nih.gov/ under the accession no. MZ981768. The associated BioProject, SRA, and BioSample numbers are PRJNA799756, SRP356349, and SAMN25208295, respectively.

## References

[CIT0001] Franck P, Garnery L, Celebrano G, Solignac M, Cornuet JM. 2000. Hybrid origins of honeybees from Italy (*Apis mellifera ligustica*) and Sicily (*A. m. sicula*). Mol Ecol. 9(7):828–921. 10.1046/j.1365-294x.2000.00945.x 10886654

[CIT0002] Garnery L, Solignac M, Celebrano G, Cornuet J-M. 1993. A simple test using restricted PCR-amplified mitochondrial DNA to study the genetic structure of *Apis mellifera L*. Experientia. 49(11):1016–1021.

[CIT0003] Henriques D, Chávez-Galarza J, Quaresma A, Neves CJ, Lopes AR, Costa C, Costa FO, Rufino J, Pinto MA. 2019. From the popular tRNA leu-COX2 intergenic region to the mitogenome: insights from diverse honey bee populations of Europe and North Africa. Apidologie. 50(2):215–229.

[CIT0004] Kumar S, Stecher G, Li M, Knyaz C, Tamura K. 2018. MEGA X: molecular evolutionary genetics analysis across computing platforms. Mol Biol Evol. 35(6):1547–1549. 29722887 10.1093/molbev/msy096 PMC5967553

[CIT0005] Longo S. 1984. L’apicoltura in Sicilia orientale. Stato Attuale e Prospettive di Viluppo Apitalia. 1984:1–7.

[CIT0006] Meng G, Li Y, Yang C, Liu S. 2019. MitoZ: a toolkit for animal mitochondrial genome assembly, annotation and visualization. Nucleic Acids Res. 47(11):e63. 30864657 10.1093/nar/gkz173 PMC6582343

[CIT0007] Muñoz I, Dall’Olio R, Lodesani M, De la Rúa P. 2014. Estimating introgression in *Apis mellifera siciliana* populations: are the conservation islands really effective? Insect Conserv Divers. 7(6):563–571.

[CIT0008] Ruttner F. 1988. Biogeography and taxonomy of honeybees. Berlin (Germany): Springer.

[CIT0009] Sambrook J, Fritsch EF, Maniatis T. 1989. Molecular cloning: a laboratory manual. Vol 2. Cold Spring Harbor (NY): Cold Spring Harbor Laboratory Press.

